# Genomic Investigation of *Proteus mirabilis* Isolates Recovered From Pig Farms in Zhejiang Province, China

**DOI:** 10.3389/fmicb.2022.952982

**Published:** 2022-07-07

**Authors:** Xiaoyun Qu, Jie Zhou, Haoqi Huang, Wen Wang, Yingping Xiao, Biao Tang, Hanlin Liu, Chenggang Xu, Xingning Xiao

**Affiliations:** ^1^Key Laboratory of Zoonosis Prevention and Control of Guangdong Province, College of Veterinary Medicine, South China Agricultural University, Guangzhou, China; ^2^State Key Laboratory for Managing Biotic and Chemical Threats to the Quality and Safety of Agro-Products, MOA Laboratory of Quality & Safety Risk Assessment for Agro-Products (Hangzhou), Institute of Agro-Product Safety and Nutrition, Zhejiang Academy of Agricultural Sciences, Hangzhou, China

**Keywords:** antimicrobial resistance, biofilm information, whole genome sequencing, virulence factor, *Proteus mirabilis*

## Abstract

*Proteus mirabilis* is a common opportunistic zoonotic pathogen, and its ongoing acquisition of antimicrobial resistance genes poses challenges to clinical treatments. Human-sourced whole genomic sequencing of human *P. mirabilis* isolates has been reported, but pig-sourced isolates have not been thoroughly investigated even though these animals can serve as reservoirs for human infections. In the current study, we report a molecular epidemiological investigation to unravel the antimicrobial and virulence gene risk factors for *P. mirabilis* contamination in 9 pig farms in 3 different cities in Zhejiang Province, China. We collected 541 swab samples from healthy pigs and 30 were confirmed as *P. mirabilis*. All 30 isolates were resistant to tetracyclines, macrolides, sulfonamides, β-lactams and chloramphenicol, and all were multiple drug-resistant and 27 were strong biofilm formers. Phylogenetic analyses indicated these 30 isolates clustered together in 2 major groups. Whole genome sequencing demonstrated that the isolates possessed 91 different antimicrobial resistance genes belonging to 30 antimicrobial classes including *rmtB, sul1, qnrS1, AAC(6′) − Ib − cr, blaCTX − M − 65* and *blaOXA − 1.* All isolates contained mobile genetic elements including integrative conjugative elements (ICEs) and integrative and mobilizable elements (IMEs). Minimum inhibitory concentration (MIC) testing indicated direct correlates between cognate genes and antimicrobial resistance. We also identified 95 virulence factors, almost all isolates contained 20 fimbrial and flagellar operons, and this represents the greatest number of these operon types found in a single species among all sequenced bacterial genomes. These genes regulate biofilm formation and represent a confounding variable for treating *P. mirabilis* infections. Our *P. mirabilis* isolates were present in healthy animals, and multiple drug resistance in these isolates may serve as a reservoir for other intestinal and environmental Enterobacteriaceae members. This prompts us to more strictly regulate veterinary antibiotic use.

## Introduction

*Proteus mirabilis* is an Enterobacteriaceae member and a significant opportunistic and food-borne pathogen and second only to enteropathogenic *Escherichia coli* (EPEC) in prevalence for urinary tract infection. *P. mirabilis* can exist in a variety of environments including the intestinal tracts of humans and wild and domestic animals and can cause infections in immuno-compromised hosts resulting in diarrhea, urinary tract infection (UTI) and keratitis (Mobley and Belas, 1995; Drzewiecka, 2016; Gong et al., 2019). Importantly, antimicrobial resistance of *P. mirabilis* has become a serious impediment to clinical treatment and resistance to colistin, nitrofurans, tigecycline, tetracycline, and β-lactams have been documented (Stock, 2003).

China is the largest global antibiotic producer and consumer and more than half are used to treat food animals (Gilbert, 2012). Livestock farms are environments with high bacterial loads and high antimicrobial selective pressures; a combination favoring development of resistant bacteria (Klein et al., 2018; Qian et al., 2018). There is abundant direct and indirect evidence that the antibiotic use on farms correlates with the rise and spread of associated antibiotic resistance genes (ARG) in human pathogens and even the direct transfer of antibiotic-resistant bacteria from animals to humans (Marshall and Levy, 2011). Pigs are major meat source worldwide, especially in China and ARGs are increasingly detected in bacterial isolates from pig feces implicating pig husbandry in ARG development and spread (Larson, 2015; Shi et al., 2021). In particular, mobile genetic elements (MGE) including insertion sequences (IS), resistance plasmids, gene castle, transposons, integrative conjugative elements (ICE), and integrative and mobilizable elements (IME) allow ARG movement within or between DNA molecules. These MGE are now frequently detected in *P. mirabilis* isolates (Shelenkov et al., 2020; Wang et al., 2021).

*P. mirabilis* is also a biofilm former allowing its engraftment on living or abiotic surfaces and complicates disease treatment and enhances viability through antimicrobial and disinfectant resistance. Treatment of UTIs due to indwelling catheters can be complicated through biofilm formation that prolong bacterial presence and can lead to bladder and kidney infections that may progress to bacteremia or sepsis (Høiby et al., 2011). *P. mirabilis* contains numerous virulence factors related to adhesion, colonization, biofilm formation and pathogenicity. These include the MR/P and PMF fimbriae and the uroepithelial cell adhesin (UCA) and all can combine to enable urinary cell attachment and biofilm formation.

The emergence of multidrug-resistant (MDR) bacterial pathogens is now considered a public health risk and sources of these pathogens have been found in humans, livestock, wild animals and food. Importantly, molecular typing studies have identified animal strains that were transmitted to humans (Reich et al., 2013; Olonitola et al., 2015; Lei et al., 2016). Previously reported 7.07% *P. mirabilis* from broiler chicken samples (Li et al., 2022) and pig operations in China are currently experiencing large outbreaks of diarrheal disease in piglets (Li, 2021). As a pathogen that can cause diarrhea, *P. mirabilis* has not attracted enough attention. Therefore, the objectives were to investigated the prevalence, antimicrobial resistance and virulence genes for *P. mirabilis* to characterize risk factors of *P. mirabilis* contamination in Zhejiang Province, China. Whole genome sequencing and bioinformatics analyses are cost-effective methods for the investigation and characterization of zoonotic pathogens (Biswas et al., 2020; Liu et al., 2020, 2021). We conducted a study on 9 farms in 3 different cities in Zhejiang Province, China, which were rarely reported, to obtain *P. mirabilis* isolates from pig swab sources. The recovered strains were subjected to whole genome sequencing followed by *in silico* analysis to identify antimicrobial resistance and virulence genes. We also determined antibiotic MICs to correlate with ARG presence.

## Materials and Methods

### Sample Collection and Characterization of *Proteus mirabilis*

A total of 541 pig swab specimens were obtained from 9 farms located in 3 different cities in Zhejiang Province, China from May to December 2021. The strains isolated from Jinhua, Hangzhou, and Quzhou were designated PM 1 (*n =* 101), PM 2 (*n =* 240), and PM 3 (*n =* 200), respectively (Table 1). In brief, swabs were placed in sterile centrifuge tubes containing 3 ml Selenite Cystine Broth (SC; Hopebiol, Qingdao, China) and incubated at 37°C for 12 h. Loopfuls were then streaked on Salmonella-Shigella agar plates (SS; Becton Dickinson, Franklin Lakes, NJ, United States) and incubated as per above (Li et al., 2022). Suspected *P. mirabilis* colonies were streak-purified a second time. Presumptive *P. mirabilis* colonies displayed black centers with transparent edges on SS agar.

**Table 1:** Sampling design and prevalence of *Proteus mirabilis* from different sources.

Sources		No. of samples	Group	No. of positive	Percentage of isolates
City	Farm				
Jinhua	1	31	PM1	2 (6.45%)	8.91%
	2	33		4 (12.12%)	
	3	38		3 (7.89%)	
Hangzhou	4	78	PM2	6 (7.69%)	4.58%
	5	80		3 (3.75%)	
	6	82		2 (2.44%)	
Quzhou	7	63	PM3	5 (7.94%)	5.00%
	8	74		3 (4.05%)	
	9	63		2 (3.17%)	
Total		541		30	5.55%

### Identification and Phylogenetic Analyses of *Proteus mirabilis*

Suspected *P. mirabilis* were selected for genomic DNA extraction using a TIANamp bacteria DNA kit (Tiangen Biotech, Beijing, China) according to the instructions of the manufacturer and DNA was stored at −20°C. *P. mirabilis* identities were confirmed using PCR detection of the *atpD* gene (5′-AGAGTTTGATCCTGGCTCAG-3′5′-ACGGGCGGTGTGTRC-3′) as previously described (Bi et al., 2013). In addition, the 16S rDNA genes (5′-AGAGTTTGATCCTGGCTCAG-3′5′-ACGGGCGGTGTGTRC-3) from these isolates were sequenced. Pure cultures of identified strains were cryopreserved at −80°C in 30% glycerol.

### Biofilm Formation Ability

Biofilm formation was assayed using the crystal violet (CV) staining method as previously described (Stepanović et al., 2007; Han et al., 2015; Lajhar et al., 2018). Briefly, bacterial suspension turbidities were adjusted to 0.5 McFarland standard (~10^8^ CFU/ml) and 20 μl was then added to prepared microtiter plates containing 180 μl Luria Bertani (LB) broth per well; negative control wells contained only LB. The plates were then covered and incubated for 24 h at 37°C under static conditions. The liquid was then discarded and the wells were washed 3× with phosphate-buffered saline (PBS) and 200 μl 0.1% CV (Sigma, Aldrich, United States) was then added per well and the plates were allowed to stand for 30 min at room temperature. The CV was decanted and the bacteria were then heat-fixed by exposure to a stream of hot air at 60°C for 60 min. The dye bound to the cells was then resolubilized using three rinses with 200 μl of 95% ethanol per well. The optical density (OD) of combined washes was measured at 595 nm using a microtiter plate reader. The average OD_595_ values were calculated for triplicates and the tests were repeated three times.

### Genomic Sequencing and Bioinformatic Analysis

Illumina pair-end sequencing of each strain utilized 1 μg genomic DNA for library construction. The qualified library was used for Illumina NovaSeq 6,000 sequencing (150 bp * 2) at Shanghai Biozeron. The raw paired end reads were trimmed and quality controlled by Trimmomatic version 0.36. ABySS^1^ was used for genome assembly with multiple-Kmer parameters for optimization. GapCloser^2^ was subsequently applied to fill remaining local inner gaps and correct single base polymorphisms for the final assembly results. *Ab initio* prediction methods were used to obtain gene models for *P. mirabilis* strains and were identified using GeneMark and then used for BLASTp searches against the non-redundant (NR) NCBI database. Potential virulence factors were assessed by searches against the Virulence Factors of Pathogenic Bacteria Database (VFDB) and BLAST results were considered significant at E < 1e^−5^ and is a generally accepted consensus cut-off (Chen et al., 2005). ARGs of the 30 *P. mirabilis* strains we sequenced were also examined using the Comprehensive Antibiotic Resistance Database (CARD), and ICEs were identified using ICEfinder (Liu et al., 2019). The public data of reference genome of *P. mirabilis* (HI4320) was obtained from NCBI^3^; gene annotation information including functional annotations and gene family information were also downloaded. The datasets generated for this study can be found in the NCBI Bioproject with the accession number no. PRJNA841796.

### Phenotypic Antimicrobial Resistance Testing

Broth dilution method was used for MIC test for a panel of 15 antimicrobial agents belonging to 9 classes as described previously (Kobylka, Kuth, Müller, Geertsma, & Pos, 2020). MIC results were interpreted according to the recommendations of the Clinical Laboratory Standard Institute guidelines (CLSI, 2017; Table 2). The antimicrobial agents were grouped in the following classes: tetracyclines (tetracycline, TET, 8–256 μg/ml; minocycline, MIN, 4–256 μg/ml), fluoroquinolones (ofloxacin, OFX, 2–64 μg/ml; nalidixic acid, NAL, 2–64 μg/ml; ciprofloxacin, CIP, 2–64 μg/ml), macrolides (azithromycin, AZ, 16–512 μg/ml; erythromycin, EM, 16–512 μg/ml), β-lactamase inhibitors (amoxicillin/clavulanic acid, AMC, 4/2–128/64 μg/ml), penicillins (ampicillin, AMP, 16–512 μg/ml), carbapenems (meropenem, MEM, 1–64 μg/ml), cephalosporins (ceftiofur, CEF, 16–512 μg/ml), aminoglycosides (amikacin, AMK, 2–64 μg/ml; gentamicin, GEN, 2–128 μg/ml), phenicols (chloramphenicol, CHL, 4–128 μg/ml; rifampicin, RIF, 1–64 μg/ml).

**Table 2:** Antimicrobial susceptibility tests for *P. mirabilis* strains used in this study (*n* = 30).

Antimicrobial drug		Abbreviation	Breakpoint (μg/ml)			Results (%)		
Species	Name		*S*	*I*	*R*	*S*	*I*	*R*
**Tetracyclines**								
Tetracycline		TET	≤4	8	≥16	0% (0/30)	0% (0/30)	100.00% (30/30)
Minocycline		MIN	≤4	8	≥16	0% (0/30)	0% (0/30)	100.00% (30/30)
**Fluoroquinolones**								
Ofloxacin		OFX	≤2	4	≥8	30.00% (9/30)	26.67% (8/30)	43.33% (13/30)
Naphthaleneic acid		NAL	≤16	–	≥32	26.67% (8/30)	0% (0/30)	73.33% (22/30)
Ciprofloxacin		CIP	≤0.25	0.5	≥1	23.33% (7/30)	0% (0/30)	76.67% (23/30)
**Macrolide**								
Azithromycin		AZ	≤2	4	≥8	0% (0/30)	0% (0/30)	100.00% (30/30)
Erythromycin		EM	≤0.5	1	≥8	0% (0/30)	0% (0/30)	100.00% (30/30)
**β-Lactam combinations**								
Ampicillin		AMP	≤8	–	≥16	10.00% (3/30)	0% (0/30)	90.00% (27/30)
Amoxicillin/clavulanic acid		AMC	≤8/4	–	≥16/8	10.00% (3/30)	0% (0/30)	90.00% (27/30)
Ceftiofur		CEF	≤2	4	≥8	16.67% (5/30)	6.67% (2/30)	76.67% (23/30)
Meropenem		MEM	≤1	2	≥4	40.00% (12/30)	60.00% (18/30)	0% (0/30)
**Aminoglycosides**								
Amikacin		AMK	≤16	32	≥64	70.00% (21/30)	10.00% (3/30)	20.00% (6/30)
Gentamicin		GEN	≤4	8	≥16	30.00% (9/30)	13.33% (4/30)	56.67% (17/30)
**Phenicols**								
Chloramphenicol		CHL	≤8	16	≥32	0% (0/30)	0% (0/30)	100.00% (30/30)
**Rifamycin**								
Rifamycin		RIF	≤1	2	≥4	26.67% (8/30)	33.33% (10/30)	40.00% (12/30)

*Proteus mirabilis* CMCC 49005 was used for quality control.

### Data Analysis

GraphPad Prism 8 software (San Diego, CA, United States) was used for figure generation. ANOVA was performed using least squares techniques with IBM SPSS Statistics 20 software (SPSS, Chicago, IL, United States). A significant difference was established at *p* < 0.05. MIC results of intermediate susceptibility were merged with resistance and each test result (resistant or susceptible) was compared with the detection (presence or absence) of the corresponding ARG *in silico*.

## Results and Discussion

*Proteus mirabilis* has been frequently incriminated in food-borne and urinary tract infections in humans. We first explored the prevalence and characteristics of *P. mirabilis* in pig farm isolates from Zhejiang province, China.

### *Proteus mirabilis* Prevalence

**Figure 1:**
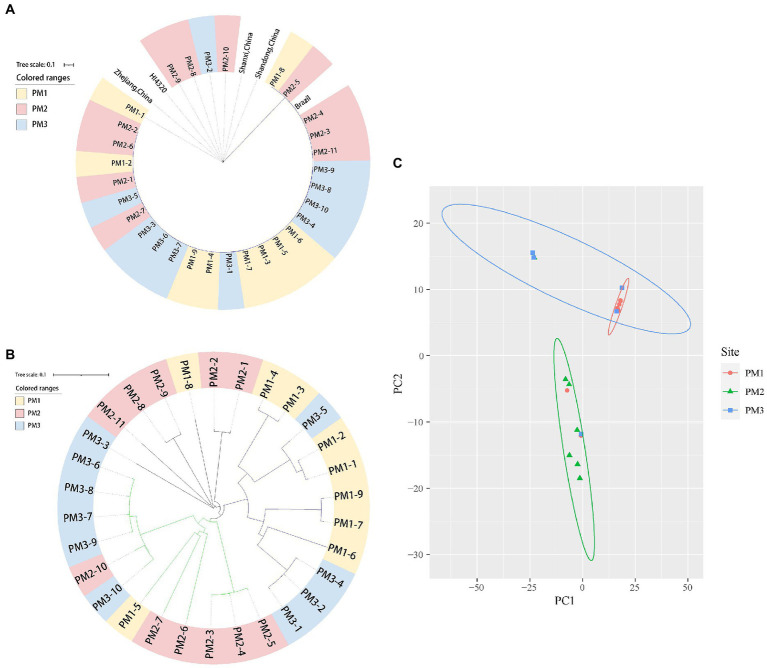
Phylogenetic tree of 31 *P. mirabilis* isolates in this study. **(A)** 16S rDNA tree. **(B)** SNP tree. **(C)** PCA analysis.

We isolated 30 *P. mirabilis* strains from 541 samples (5.55%) using *atpD* PCR detection, 16S rDNA and whole genome sequencing (Table 1). The PM1 group (Jinhua) was the most contaminated (8.91%), and this level was close to that previously reported (7.07%) from broiler chicken samples (Li et al., 2022) but lower than from human sources (Mirzaei et al., 2019; Tabatabaei et al., 2021). Phylogenetic analysis identified 2 major clusters including Group 1 that contained reference strain LR738973_1 from fecal samples of weaned piglets in Brazil and the smaller Group 2 that included model strain HI4320, MK758055.1 from human feces, AM231709.1 from fish bowels and OL629222.1 from raccoon feces in China (Figure 1A). The isolates were also clustered closely in the phylogenetic analysis of core SNPs especially between PM1 and PM3 (Figure 1B), and PCA analysis indicated PM 1 and PM 3 were closely linked (Figure 1C), which might because Jinhua and Quzhou are closer together.

### Biofilm Information Ability

**Figure 2:**
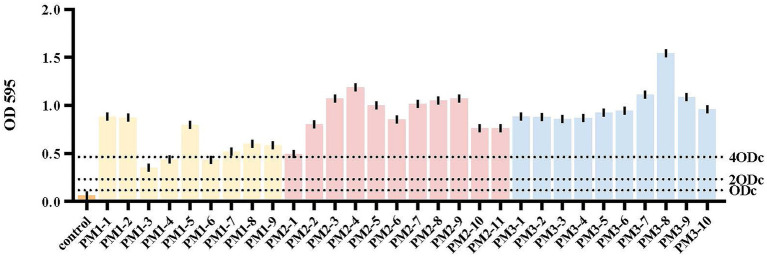
Biofilm formation. Yellow, PM1; pink, PM2; blue, PM3. The cut-off value (ODc) was defined as 3 SD above the mean OD_595_ of the negative control: ODc = average OD_595_ of negative control + (3 × SD of negative control). OD_595_ ≤ ODc = absence of biofilm; ODc ≤ OD_595_ ≤ 2 × ODc = weak biofilm producer; 2 × ODc ≤ OD_595_ ≤ 4 × ODc = moderate biofilm producer; 4 × ODc ≤ OD_595_ = strong biofilm producer.

The 30 *P. mirabilis* isolates we identified were also biofilm formers (Figure 2). For example, isolates PM1-3, PM1-4 and PM1-6 displayed a moderate biofilm formation capacity (0.2314 ≤ OD_595_ ≤ 0.4628) while 27 isolates were strong biofilm formers (OD_595_ ≥ 0.4628). All PM2 and PM3 isolates were in this biofilm formation capacity category and significantly different from isolates from PM1 (*p* < 0.05). Bacterial biofilms are resistant to antibiotics, disinfectants and phagocytosis as well as other components of innate and adaptive inflammatory systems (Høiby et al., 2011). Compared with planktonic cells, biofilm cells are less sensitive to antimicrobial agents and limit antibiotic entry to low levels (Tseng et al., 2013). In this study, 90% (27/30) of our isolates were strong biofilm formers and these could be ranked as PM3 > PM2 > PM1. Interestingly, this ranking was consistent with the MIC results (see below) and intensity of biofilm formation has been previously linked to antibiotic resistance (Mirzaei et al., 2019) and MDR strains are often biofilm formers, similar to our findings (Sanchez et al., 2013).

### Phenotypic Antimicrobial Resistance

MIC testing of our 30 isolates indicated different degrees of resistance to the 15 antimicrobials we analyzed. *P. mirabilis* is naturally resistant to tetracycline, tigecycline, and polymyxin, but sensitive to carbapenems and aminoglycosides although amikacin and meropenem resistance has been increasing and is linked with their abuse (Kang et al., 2021). Our results were consistent with these observations and most isolates were sensitive to the aminoglycoside amikacin (21/30) and the carbapenem meropenem (12/30). These levels were lower than those found in two recent hospital studies in Iran that documented 71% and 82% and 82% and 82%, respectively (Mirzaei et al., 2019; Tabatabaei et al., 2021), and higher than the 10.5% sensitive to meropenem in a Chinese hospital (Hu et al., 2012; Han et al., 2015). All our strains were resistant to the tetracyclines (tetracycline, minocycline), macrolides (azithromycin, erythromycin) and chloramphenicol (chloramphenicol) classes and to a lesser extent to amoxicillin/clavulanic acid (27/30), ampicillin (7/30), ciprofloxacin (23/30), and rifampicin (22/30; Table 2). Rifampicin is a broad-spectrum antibiotic used primarily in clinical treatment of tuberculosis (Chen et al., 2017). Chloramphenicol was forbidden for veterinary use in China in 2002 (The Ministry of Agriculture of the People’s Republic of China, 2002) so it is intriguing why we found such high levels of resistance to these drugs.

**Figure 3:**
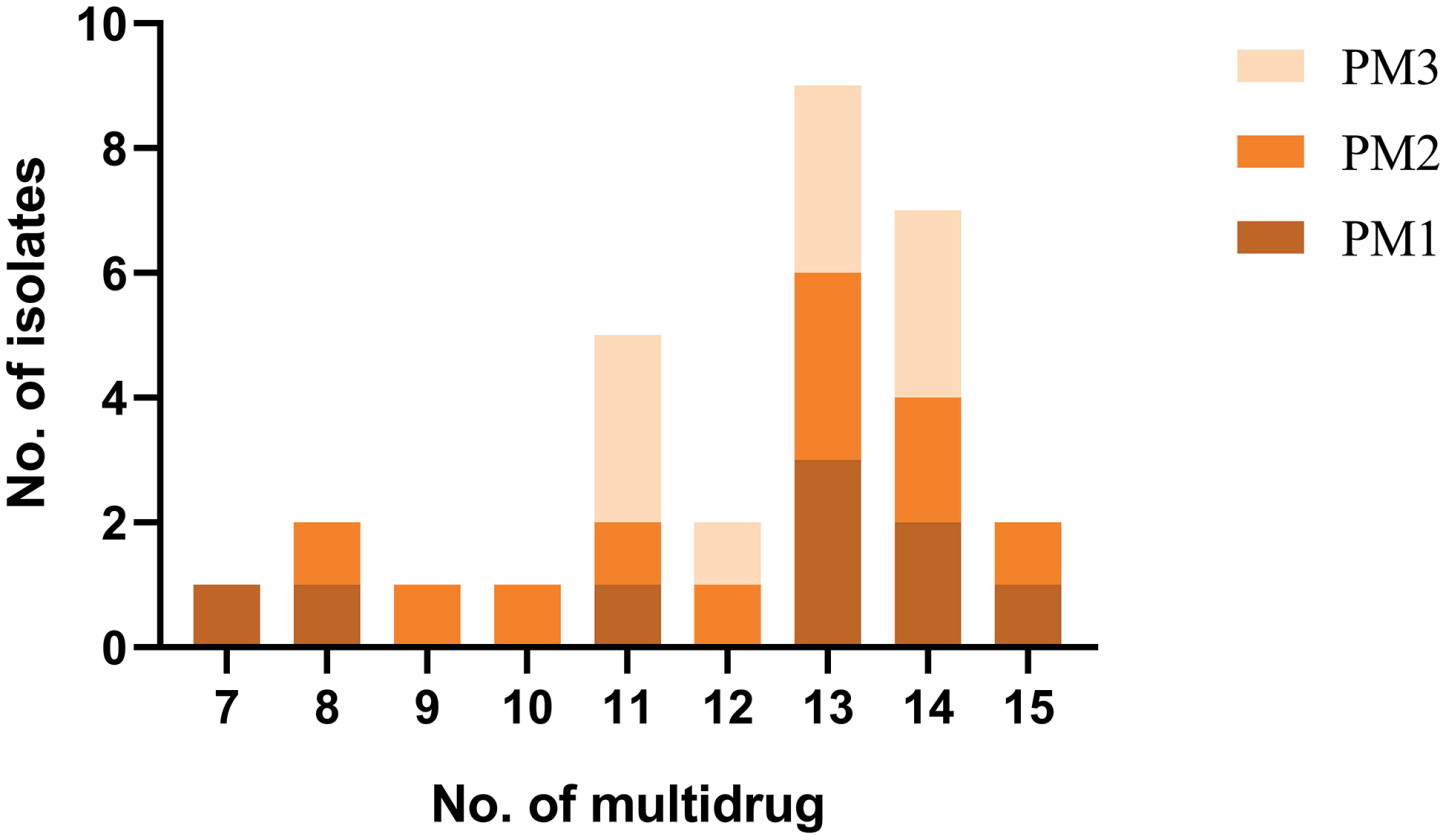
MDR tests of *P. mirabilis* isolates.

**Figure 4:**
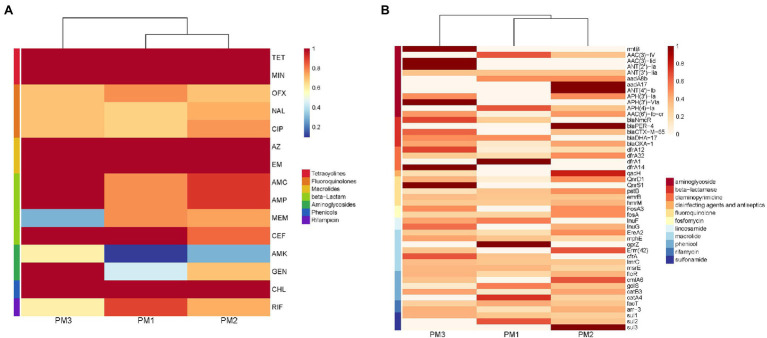
Heatmap of antimicrobial resistance of *P. mirabilis* isolates according to **(A)** phenotype and **(B)** genotype.

Our PM3 group displayed the highest prevalence for antibiotic resistance to amoxicillin/clavulanic acid, ampicillin, gentamycin and amikacin (Figure 4A). Moreover, all our isolates were resistant to at least 3 antimicrobial classes and were considered MDR (Figure 3). These data were similar to previous findings of resistance in animals in China (Li et al., 2022). These high levels of drug resistance and prevalence of MDR *P. mirabilis* on these farms pose a grave public health threat and would render clinical treatment protocols problematic.

### ARG Predictions

Genomic analysis identified the presence of 91 ARGs in our isolates that encoded resistance to 30 antimicrobial classes. The most prevalent (total 43 genes) resistance mechanisms were antibiotic efflux pumps (42.86%), antibiotic inactivation (27.47%), antibiotic target alteration (15.38%), antibiotic target protection (6.59%) and antibiotic target replacement (7.69%). Interestingly, 43 (47.25%) ARGs were present in all isolates. These ARGs included members of the ATP-binding cassette (ABC), major facilitator superfamily (MFS), *pmr* phosphoethanolamine transferase, resistance-nodulation-cell division (RND), glycopeptide resistance gene cluster, *Acinetobacter* mutant *lpx* gene conferring resistance to colistin and ABC-F ATP-binding cassette ribosomal protection protein MDR gene family members *bcr-1*, *tet(J)*, *tetA(48)*, *msbA*, *mdtB*, and *mexA* (Supplementary Table S1).

**Figure 5:**
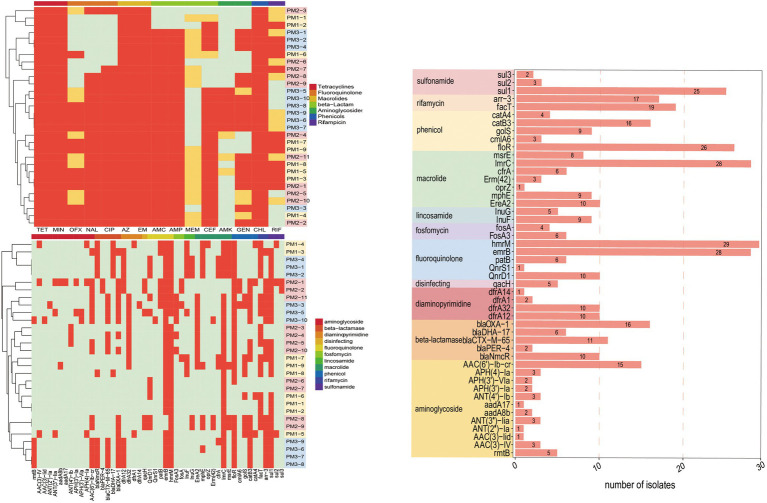
ARGs present in *P. mirabilis* genomes of study isolates. **(A)** Phenotype; PM 1–5, PM 2–12 were resistant to all the tested drugs. **(B)** Genotype, excepting ARGs that all strains contained. **(C)** Fluoroquinolone, macrolide, phenicol, and sulfonamide prevalence. Red, positive; green, negative.

The remaining 48 ARGs were variably present among the isolates. These were associated with different antibiotic classes: *blaCTX-M-65*, *blaDHA-17*, *blaNmcR*, *blaOXA-1*, *blaPER-4*, and *blaPER-4* for β-lactams; *sul1*, *sul2*, *sul3* for sulfonamides; *rmtB* and AAC, ANT, APH groups for aminoglycosides, *dfr*A groups for trimethoprim, *lnu* for lincosamide, e*reA2*, *mphE*, *emrB* for macrolides; *qnrD1*, *qnrS1* for quinolones; *fos* for fosfomycin; *fact*, *arr-3* for rifamycin and *cml*, *flo*R, *cat* for phenicols. In particular, >50% of these isolates contained *hmrM*, *emrB*, *ImrC*, *floR*, *sul1*, *fact*, *arr-3*, *blaOXA-1*, *catB3* and *AAC(6′)-Ib-cr* that encode resistance to fluoroquinolones, macrolides, phenicols, sulfonamides, rifamycin, aminoglycosides, and β-lactams, respectively. We also detected the *qacH* gene in 5 isolates; this gene encodes resistance to disinfecting agents and antiseptics (Figure 5C).

The extended-spectrum β-lactamases (ESBL) were reported for the first time in 1983 (Knothe et al., 1983) and *P. mirabilis* is highly represented as an ESBL host. These representatives include the cephalosporinase (AmpC) and TEM and CTX-M type carbapenemases that can degrade penicillin, piperacillin, and the cephalosporins (Bontron et al., 2019). We found the presence of blaCTX-M in our *P. mirabilis* isolates that enables resistance to ceftiofur while piperacillin and cephalosporin resistance was linked to the presence of *blaOXA-1* gene (Algammal et al., 2021). In addition, both *blaOXA-1* and *blaCTX-M* genes synergistically enable *P. mirabilis* to resist β-lactams combination treatments (Schultz et al., 2015). In the current study, our isolates displayed a high prevalence of *blaOXA-1* (53.33%), *blaCTX-M-65* (36.67%), *blaNmcR* (33.33%), *blaDHA-17* (20%), and *blaPER-4* (13.33%) that are β-lactams resistance gene (Figure 5C). Remarkably, 10 isolates contained both *blaOXA-1* and *blaCTX-M-65* and 7 in PM3 that also displayed the highest phenotypic resistance to the β-lactams ceftiofur, ampicillin, and amoxicillin/clavulanic acid (Supplementary Material; Figure 3A). The primary aminoglycoside ARG carried by all isolates in our study was *AAC (6′)-lb-cr* (50%) and 5 *rmtB* 16S rRNA methyltransferases (*rmt*) were detected only in PM3. Resistance to amikacin was also prevalent in this group (Figure 4A). The *rmtB* gene is the most frequently detected ARG in Enterobacteriaceae from humans and animals and mediates a very high-level resistance to aminoglycosides including amikacin and is usually associated with MDR bacteria (Fang et al., 2019) and was consistent with our results. We also detected a large number of tetracycline, macrolide, peptide and fluoroquinolone resistance genes and PM3 displayed resistance to tetracycline, macrolide and phenicols (Figures 4A,B). Furthermore, we evaluated the relationship between phenotypic drug resistance and the presence or absence of corresponding ARGs. All the isolates were resistant to tetracycline, minocycline, azithromycin, erythromycin, and chloramphenicol ARG, and 76.67% resistant to ciprofloxacin, ceftiofur, and gentamicin, and 60% to meropenem and possessed the cognate ARGs. However, genotypic and phenotypic resistance are sometimes not directly linked although the drug-resistant phenotypes and genotypes of most bacteria were consistent (Table 3). Eight strains did not contain any β-lactamase resistance and only one (PM2-3) was sensitive to all the β-lactams, 6 were partially sensitive and PM1-9 displayed resistance similar to the other 22 isolates. For rifampicin, fluoroquinolone, and aminoglycoside resistance, we could not identify any significant correlation between genotype and phenotype and resistance may be mediated by efflux pump mechanisms (Supplementary Table S2).

**Table 3:** Phenotypic and genotypic analyses of antimicrobial resistance of *P. mirabilis* isolates.

Antimicrobial drugs		Coherent results		Incoherent results		Percentage of coherence
Species	Name	Both susceptible	Both resistant	Phenotype resistance and genotype susceptible	Phenotype susceptible and genotype resistance	
**Tetracyclines**						
Tetracycline		0	30	0	0	100.00% (30/30)
Minocycline		0	30	0	0	100.00% (30/30)
**Fluoroquinolones**						
Ofloxacin		0	21	0	9	70.00% (21/30)
Naphthaleneic acid		0	22	0	8	73.33% (22/30)
Ciprofloxacin		0	23	0	7	76.67% (23/30)
**Macrolide**						
Azithromycin		0	30	0	0	100.00% (30/30)
Erythromycin		0	30	0	0	100.00% (30/30)
**β-Lactam combination**						
Ampicillin		3	17	9	0	70.00% (21/30)
Amoxicillin/clavulanic acid		3	17	9	0	70.00% (21/30)
Ceftiofur		3	20	5	2	76.67% (23/30)
Meropenem		4	14	4	8	60.00% (18/30)
**Aminoglycosides**						
Amikacin		10	9	0	11	63.33% (19/30)
Gentamicin		6	17	4	3	76.67% (23/30)
**Phenicols**						
Chloramphenicol		0	30	0	0	100.00% (30/30)
**Rifamycin**						
Rifamycin		0	19	3	8	63.33% (19/30)

Our results indicated that the distribution of drug-resistant phenotypes was correlated with genotypes for β-lactams and aminoglycosides although these differed by degree. These may due to the complex drug resistance mechanisms in agreement with a study indicating that the phenotypic test and not possession of a specific ARG is the gold standard for assessment of bacterial drug resistance (Wu et al., 2021).

### MGE Prediction

The presence of MGEs in *P. mirabilis* are a great public health threat due to the capacity for autonomous ARG transfer (Partridge et al., 2018). ICEs are integrated DNA regions in the chromosome that are mobilizable by conjugation *via* type IV secretion systems that mediate cell to cell DNA transfer (Delavat et al., 2017). Tyrosine and serine recombinases can mediate *att* site-specific recombination between circular ICE and chromosomal targets (Johnson and Grossman, 2015). IMEs can be mobilized using ICE machinery or conjugative plasmids when ICEs erode and accumulate within the host chromosome leading to inactivation of their independent mobility (Partridge et al., 2018).

In our study, we identified 15 ICEs and 7 IMEs and 53.33% of our isolates contained ICEs or IMEs with 13 of 30 containing ICEs and 6 of 30 containing IMEs (Table 4). These results were higher than previously reported (23.53%) in *P. mirabilis* from the tree shrew (Gu et al., 2020). Most MGEs were detected in PM3 (90%) and 2 PM1 strains possessed the same ICE type (Table 5). This indicated that transconjugation was frequent for this group. Moreover, strains with ICEs from the same group carried similar resistance genes especially in PM1 and PM3. All of the PM1 strains with MGEs (PM1-7, PM1-8, PM1-9) were clustered. Strains with MGEs in PM3 gathered into 3 small groups and carried the same resistance gene (PM3-8, PM3-9 and PM3-1, PM3-2, PM3-4; Figure 5 and Table 5). Such results indicate the serious drug resistance of PM3 group may be associated with MGEs. The high MDR prevalence could be attributed to a combination of these factors. However, we did not perform a validation analysis of these detected MGE functions and further analyses are needed to understand the effect of these elements on drug resistance.

**Table 4:** MGE prediction for the 30 isolates examined in this study.

Group	Isolates	MGE numbers		Number positive	
		ICE	IME	ICE	IME
PM1 (*n* = 9)	PM1-7	1	0	33.33% (3/9)	0.00 (0/9)
	PM1-8	2	0		
	PM1-9	1	0		
PM2 (*n* = 11)	PM2-3	0	1	18.18% (2/11)	36.36% (4/11)
	PM2-5	0	1		
	PM2-6	1	2		
	PM2-10	1	1		
PM3 (*n* = 10)	PM3-1	2	0	80.00% (8/10)	20.00% (2/10)
	PM3-2	1	0		
	PM3-3	1	0		
	PM3-4	1	0		
	PM3-5	1	1		
	PM3-6	1	0		
	PM3-8	0	1		
	PM3-9	1	0		
	PM3-10	1	0		
Total	53.33% (16/30)	15	7	43.33% (13/30)	20.00% (6/30)

**Table 5:** Details of 15 predicted ICEs and 7 IMEs.

Isolate	Location (nt)	GC content (%)	Length (bp)	Direct repeats	Type
PM1-7	1809473..1902135	44.85	92,663		Putative ICE with T4SS
PM1-8	2873874..2966537	44.85	92,664	^†^	Putative ICE with T4SS
	3727325..4093850	41.37	366,526	attL: 3727325..3727339 (agaggtcattgtgca) attR: 4093836..4093850 (agaggtcattgtgca)	Putative ICE with T4SS
PM1-9	1810225..1902887	44.85	92,663	^≠^	Putative ICE with T4SS
PM2-6	1741700..1935382	41.39	193,683	attL: 1741700..1741714 (ttttgaatgacataa) attR: 1935368..1935382 (ttttgaatgacataa)	Putative ICE with T4SS
PM2-10	3589109..3824596	40.54	235,488	attL: 3589109..3589124 (taattgccattatatt) attR: 3824581..3824596 (taattgccattatatt)	Putative ICE with T4SS
PM3-1	3795192..3926392	44.63	131,201	attL: 3795192..3795206 (agatacattttgttt) attR: 3926378..3926392 (agatacattttgttt)	Putative ICE with T4SS
	1308600..1414471	43.22	105,872	attL: 1308600..1308614 (tattgccgctttaat) attR: 1414457..1414471 (tattgccgctttaat)	Putative ICE with T4SS
PM3-2	1678276..1746776	43.40	68,501	attL: 1678276..1678290 (taaaagcaacagcat) attR: 1746762..1746776 (taaaagcaacagcat)	Putative ICE with T4SS
PM3-3	3696382..3796909	41.98	100,528	attL: 3696382..3696396 (taatgctattttttt) attR: 3796895..3796909 (taatgctattttttt)	Putative ICE with T4SS
PM3-4	1678055..1746555	43.40	68,501	attL: 1678055..1678069 (taaaagcaacagcat) attR: 1746541..1746555 (taaaagcaacagcat)	Putative ICE with T4SS
PM3-5	3524314..3652232	42.17	127,919	attL: 3524314..3524328 (ataaaatacttttta) attR: 3652218..3652232 (ataaaatacttttta)	Putative ICE with T4SS
PM3-6	3603558..3866601	40.46	263,044	attL: 3603558..3603573 (tctgtgcagtaaaaaa) attR: 3866586..3866601 (tctgtgcagtaaaaaa)	Putative ICE with T4SS
PM3-9	3601557..3864543	40.45	262,987	attL: 3601557..3601572 (tctgtgcagtaaaaaa) attR: 3864528..3864543 (tctgtgcagtaaaaaa)	Putative ICE with T4SS
PM3-10	3716853..3757384	44.69	40,532	–	Putative ICE with T4SS
PM2-3	3790440..3819943	44.38	29,504	attL: 3790440..3790454 (caaaaccataaaacc) attR: 3819929..3819943 (caaaaccataaaacc)	Putative IME
PM2-5	3363919..3384240	40.87	20,322	attL: 3363919..3363933 (ccaaaaaatgcatta) attR: 3384226..3384240 (ccaaaaaatgcatta)	Putative IME
PM2-6	3316029..3385857	36.68	69,829	attL: 3316029..3316044 (aaaatattagtgagta) attR: 3385842..3385857 (aaaatattagtgagta)	Putative IME
	3853151..3907640	45.54	54,490	^††^	Putative IME
PM2-10	3847018..3872472	42.51	25,455	attL: 3847018..3847032 (cgctgatgcagtaac) attR: 3872458..3872472 (cgctgatgcagtaac)	Putative IME
PM3-5	1510679..1556480	40.12	45,802	attL: 1510679..1510694 (cagcaatggatattta) attR: 1556465..1556480 (cagcaatggatattta)	Putative IME
PM3-8	3864568..3889007	42.45	24,440	attL: 3864568..3864582 (cgctgatgcagtaac) attR: 3888993..3889007 (cgctgatgcagtaac)	Putative IME

*^†^ attL: 2873874..2873925 (atggtgcccggactcggaatcgaaccaaggacacggggattttcaatcccct); attR: 2966486..2966537 (atggtgcccggactcggaatcgaaccaaggacacggggattttcaatcccct)*.

*^≠^ attL: 1810225..1810276 (atggtgcccggactcggaatcgaaccaaggacacggggattttcaatcccct); attR: 1902836..1902887 (atggtgcccggactcggaatcgaaccaaggacacggggattttcaatcccct)*.

*^††^ attL: 3853151..3853228 (ggattgttcacccactaatagggaacgtgagctgggtttagaccgtcgtgagacaggttagttttaccctactgatga); attR: 3907563..3907640 (ggattgttcacccaccaatagggaacgtgagctgggtttagaccgtcgtgagacaggttagttttaccctactgatga)*.

### Virulence Gene Prediction

**Figure 6:**
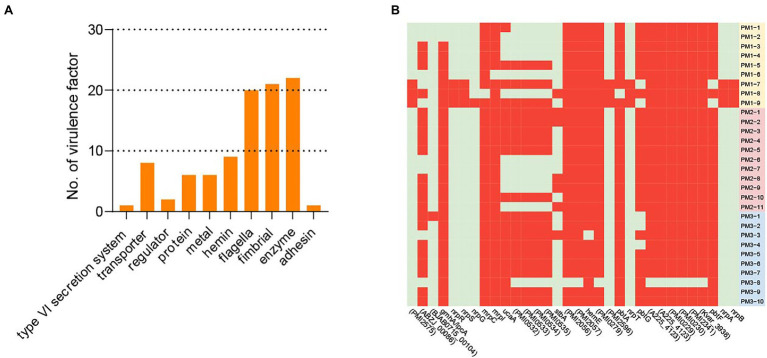
Virulence factors. **(A)** Virulence factor categories and the number represented in each category. **(B)** Virulence factors that were variably present among isolates. Red, positive; green, negative.

Most of the endemic strains in our study were MDR and the number of virulence genes identified would contribute to the establishment of persistent and drug-tolerant infections. In our 30 isolates, we identified 95 of 2,741 possible virulence factors composed of 10 categories: transporter, fimbriae, flagella, hemin, metal, enzyme, protein, regulator, type VI secretion system, and adhesin. Enzyme represented 22 virulence factors related to factors such as DP-heptose synthase, UDP-glucose 6-dehydrogenase, and decarboxylase. The flagella and fimbrial groups were also abundant and 20 different genes were represented. The least abundant were the type VI secretion system and adhesin (Figure 6).

All isolates contained 63/95 virulence factors associated with biofilm formation, adhesin, carbon storage, hemin, metal and motility and 32/95 were variably present among the 30 isolates. All strains contained at least 8 of the 32 variable virulence genes that included the categories transporter, Proteus-like (MR/P) fimbriae, protein, adhesin, enzyme, fimbrial, metal, and regulator that included ABC transporter permease, hemolysin activator protein, fimbrial adhesin, IS4 family transposase ORF 2, siderophore biosynthesis protein, and non-ribosomal peptide synthase. The PM3 group contained the largest numbers of virulence factors while 10 isolates did not contain any virulence factors related to UCA in the PM1 and PM2 groups (Figure 6B, Supplementary Table S2).

The virulence factors for the MR/P and PMF fimbriae and UCA are considered essential in the initial phase of infection and contribute significantly to UTI development (Cook et al., 1995; Jansen et al., 2004; Govindarajan and Kandaswamy, 2022). Aggregation and initial biofilm formation are mediated by MR/P fimbriae that are products of the *mrp* operon (Wasfi et al., 2020). The UCA fimbriae are controlled *via* the *ucaA* gene and PMF confers bladder colonization. All of our isolates contained PMF and 20 contained *ucaA* and most contained 5 other UCA-related virulence genes (Supplementary Table S3). Moreover, we found 20 putative fimbrial operons and this is the largest number found in any sequenced bacterial species (Scavone et al., 2016).

*P. mirabilis* is highly mobile and swarm ability allows it to pass through catheters to the urinary tract. Flagella are important to swarmer cell differentiation and are necessary to swarming motility (Belas and Suvanasuthi, 2005). In our study, we found all the isolates carried 20 different flagellar virulence factors including *flgI*, *film*, and *flgC*. Most importantly, 29 isolates except PM3-8 contained the *ZapA* virulence factor (Belas et al., 2004) that enables the bacterium to survive in the urethra *via* degradation of host proteins using the Zap1 metalloproteinase (Supplementary Table S3). Our findings indicated the *P. mirabilis* isolated from healthy pigs is a potential threat to public health since its high prevalence and possession of virulence genes and ARGs can be transferred *via* MGEs.

## Conclusion

In this study, we conducted the prevalence and characteristics of *P. mirabilis* from healthy farmed pigs in China. Phylogenetic analysis indicated the isolates were closely related and clustered into 2 major groups. Our 30 isolates harbored 91 ARGs and >50% contained MGEs as well as 95 different virulence factors that included the presence of 20 putative fimbrial operons. Most (90%) of the isolates were strong biofilm formers. This is the first study to report the genomic investigation and prevalence of *P. mirabilis* associated with pig farm operations in Zhejiang, China.

## Data Availability Statement

The datasets presented in this study can be found in online repositories. The names of the repository/repositories and accession number(s) can be found in the article/Supplementary Material.

## Author Contributions

XX and CX: writing—review and editing. XQ and JZ: investigating and writing—original draft. HH and WW: data curation. YX, BT, and HL: resources. All authors contributed to manuscript revision, read, and approved the submitted version.

## Funding

This research was supported by the Key Research and Development Program of Zhejiang Province (2022C02049), Ministry of Agriculture and Rural Affairs (14215033), and the Walmart Foundation (UA2020-152 and UA2021-247).

## Conflict of Interest

The authors declare that the research was conducted in the absence of any commercial or financial relationships that could be construed as a potential conflict of interest.

## Publisher’s Note

All claims expressed in this article are solely those of the authors and do not necessarily represent those of their affiliated organizations, or those of the publisher, the editors and the reviewers. Any product that may be evaluated in this article, or claim that may be made by its manufacturer, is not guaranteed or endorsed by the publisher.

## Footnotes

^1^ http://www.bcgsc.ca/platform/bioinfo/software/abyss

^2^ https://sourceforge.net/projects/soapdenovo2/files/GapCloser/

^3^ https://www.ncbi.nlm.nih.gov/assembly/GCF_000069965.1/
